# Health Care Workers’ Perspectives on the Barriers and Facilitators to Digital Health Technology Use to Support Symptomatic Cancer Diagnosis in Southern Africa: Qualitative Study

**DOI:** 10.2196/68412

**Published:** 2025-07-24

**Authors:** Kirsten D Arendse, Sarah Day, Bothwell Takaingofa Guzha, Tasleem Ras, Valerie Anne Sills, Natalie Tegama, Jennifer Moodley, Fiona M Walter, Suzanne E Scott

**Affiliations:** 1 Wolfson Institute of Population Health, Faculty of Medicine and Dentistry, Queen Mary University of London, Charterhouse Square, London, EC1M 6BQ, United Kingdom, 44 20 7882 3850; 2 School of Public Health, Faculty of Health Sciences, University of Cape Town, Cape Town, South Africa; 3 Department of Obstetrics and Gynaecology, Faculty of Medicine & Health Sciences, University of Zimbabwe, Harare, Zimbabwe; 4 Department of Family, Community and Emergency Care, Faculty of Health Sciences, University of Cape Town, Rondebosch, South Africa; 5 Cancer Research Initiative, Faculty of Health Sciences, University of Cape Town, Cape Town, United Kingdom; 6 The Primary Care Unit, Department of Public Health and Primary Care, University of Cambridge, Cambridge, United Kingdom

**Keywords:** Southern Africa, breast cancer, cervical cancer, colorectal cancer, digital health, digital technology

## Abstract

**Background:**

Despite improvements in early cancer diagnosis worldwide, morbidity and mortality in Southern Africa continue to rise owing to challenges with funding, sociocultural beliefs, and health care access. Digital health (“eHealth”) has the potential to expand access to health care, particularly to remote communities. However, few studies explored the use of eHealth to support symptomatic cancer diagnosis in Southern Africa.

**Objective:**

This study explored the barriers and facilitators to eHealth use by health care workers (HCWs) to support the management of people with symptoms of possible breast, cervical, or colorectal cancer in South Africa and Zimbabwe.

**Methods:**

We conducted semistructured in-depth interviews with HCWs (n=56) who managed people with symptoms of possible cancer. Interviews explored the barriers and facilitators to eHealth use and attitudes toward further adoption of eHealth. Interview schedules were guided by the sociotechnical theory, a model “designed to address the socio-technical challenges involved in design, development, implementation, use, and evaluation of eHealth.” The interviews were audio-recorded and transcribed. We used the framework method to analyze the data and developed themes that encompassed patterns and meaning in the data to answer the research question.

**Results:**

The median age of participants was 44 (IQR 34‐53) years, 38 (68%) were female, and most were nurses (n=34, 61%) or doctors (n=18, 32%). Four core themes were developed: (1) “the lack of reliable infrastructure hindered eHealth use among HCWs”; (2) “the use of personal mobile devices increased eHealth access at the expense of patient privacy and personal cost”; (3) “information, workflow integration, and access”; while eHealth improved access to information, many tools were already in use, were poorly integrated into workflow, and disrupted consultations; and (4) “digital health is expanding whether we like it or not,” which describes a spectrum of attitudes toward digital health, ranging from enthusiasm to resistance but willingness to adapt to those completely against its use. Themes from the workshops were concordant with the in-depth interview findings.

**Conclusions:**

To capitalize on the potential benefits of eHealth use among HCWs, such as to support early cancer diagnosis, infrastructural challenges must be addressed, and tools designed to meet user needs and be integrated into clinical workflow. As in many other resource-constrained settings, significant improvements in development are required for the value of eHealth to be realized in Southern Africa. Additionally, where resources such as electricity are limited, their use for eHealth needs to be weighed against use for other priorities such as operating ventilators. Furthermore, energy production in these regions is largely reliant on burning fossil fuels, and thus, the use of eHealth tools risks contributing negatively to climate change. The findings of this study can be used to guide future eHealth design or implementation strategies that are more contextually suitable.

## Introduction

Cancer is one of the leading causes of death worldwide, with the majority of the burden occurring in low- and middle-income countries [1]. Breast and cervical cancers remain the leading causes of cancer-related mortality in Southern Africa [1] despite global advancements in screening, early diagnosis, and treatment. Cancer incidence in Southern Africa is rising [1,2] owing to increased exposure to risk factors [3], an epidemiological shift to noncommunicable diseases, and an ageing population [4]. While health advancements have reduced breast and cervical cancer mortality in high-income nations [2], the effectiveness and sustainability of similar strategies in Southern Africa are limited by funding, sociocultural beliefs and behaviors, and health care access [5]. The World Health Organization highlighted screening and early diagnosis as key approaches to enable cancer control. Where resources are insufficient to address both, early diagnostic capacity should be a priority [6]. Despite this, few studies have explored opportunities for earlier detection in the region [7,8] compared to extensive research on screening.

Digital health technology use, also known as eHealth*,* is rapidly increasing in sub-Saharan Africa, where 43% of the population was subscribed to mobile services in 2022 [9], and has the potential to increase health care access. eHealth is defined as “the use of information and communications technology in support of health and health-related fields” and incorporates electronic health records, health information systems, clinical support systems, and health education [10]. The benefit of eHealth is in its potential to expand access to health care while minimizing costs, making it of interest in designing early cancer diagnostic approaches in low-resource settings. Many digital tools aimed at improving health outcomes in South Africa lack the evidence to support efficacy and feasibility [11], thus limiting the potential for further investment and expansion. While the innovative and transformative capabilities of eHealth to improve cancer health services are recognized, there is also a need to develop interventions that are underpinned by evidence-based design and have been shown to be effective and feasible through reliable research and evaluation.

mHealth (mobile health), a subset of eHealth, involves the use of mobile devices such as phones and tablets for medical and public health practice. The advantages of mHealth include widespread access to devices [12], portability, and ease of connectivity to information, particularly for people in remote locations. Various forms of eHealth and mHealth interventions are available to the public or patients and health care workers (HCWs). Public or patient-facing eHealth interventions are frequently in the form of mHealth and include SMSs and mobile apps. eHealth is increasingly being adopted in health care to support administration, clinical management, and HCW training and education [13].

This study aimed to explore the barriers and facilitators of eHealth use by HCWs to support the management of people with possible symptoms of cancer in South Africa and Zimbabwe. This includes understanding the contextual factors that could impact the implementation and uptake of eHealth and user needs that should be considered for future eHealth design or adaptation to existing tools.

## Methods

### Study Design and Reporting

A qualitative in-depth interview (IDI) study was conducted with HCWs managing patients with possible cancer symptoms. We also conducted workshops with clinicians to verify the credibility of the interview findings. The project was part of the AWACAN-ED (African Awareness of Cancer & Early Diagnosis) Program [14], which aims to advance awareness and early cancer diagnosis. The Standards for Reporting Qualitative Research Checklist [15] was used to ensure that all aspects of the qualitative design were incorporated into this study (Checklist 1).

### Study Setting

This study was conducted in 2 regions of South Africa (The Western Cape and Eastern Cape Provinces) and 2 regions in Zimbabwe (Harare and Bulawayo, including their referral provinces) across all levels of health care. The countries were selected for variation in development and resources; South Africa has a high Human Development Index (HDI) and Zimbabwe a middle HDI [16,17]. Public health care in both countries consists of 3 tiers with increasingly specialized services [18]. Primary-level facilities are clinics that provide basic health services usually during working hours, such as chronic care, antenatal services, and screening; they are the first point of contact for most patients with the health care system, and most clinical consultations are conducted by nurses. Secondary- and tertiary-level health facilities are hospitals that provide more specialized inpatient and outpatient services, including medical, surgical, and emergency services. Services offered at the secondary and tertiary levels vary across regions, and cancer diagnostic and treatment services are usually managed at the tertiary level. People presenting with possible symptoms of breast, cervical, or colorectal cancer usually present to primary care facilities and are thereafter referred to secondary or tertiary care for investigations (eg, for colposcopy, mammography, biopsy, and colonoscopy) and treatment.

### Sample

Potential participants were eligible for inclusion if they worked with patients presenting with symptoms of possible breast, cervical, or colorectal cancers. We used a matrix to purposively sample participants by region, health facility level, and job role, aiming for at least 12 primary care HCWs and at least 7 secondary or tertiary HCWs in each country.

### Procedure

We purposively sampled participants to ensure representation of all levels of care across the region. In primary care, facility managers identified potential participants across a range of roles, ages, and experience levels who met the inclusion criteria. Potential participants were provided with an information sheet, and the study was discussed in person. If they agreed to participate, we returned at a later date to conduct interviews. In secondary and tertiary care, the heads of departments were sent an information sheet via email and forwarded to staff who met the inclusion criteria. Additionally, participants sometimes recommended other potential participants. Experienced qualitative researchers (SD, Melinda Mel Moyo, MSc, and Sunga Mzeche, MSc) conducted interviews. Most interviews took place in a private setting at a health facility, while 2 took place virtually via Microsoft Teams, and all lasted 30‐90 minutes. Interviews were conducted in English or local languages (isiXhosa in South Africa and Ndebele or Shona in Zimbabwe) based on the participants’ preferences. In 1 interview, 3 HCWs were present in addition to the main interviewee; questions were directed to and answered by the main interviewee, while the additional HCWs added details. This interview was counted as 1 participant. Interviews were audio-recorded and transcribed verbatim using a professional transcription company. Interviews conducted in local languages were translated into English by the transcriber at the time of transcription. After the first few interviews in each country, debriefing was conducted, and the interview guides adapted where necessary. A sample of transcriptions was checked by the interviewers (Sunga Mzeche and Melinda Mel Moyo) to ensure accuracy and completeness. Interviews were conducted until data saturation was reached, between June 2023 and January 2024.

Interviews began with asking sociodemographic questions (age, job role, gender, education level, and years of experience). A semistructured approach was used following a topic guide and aimed to explore (1) barriers and facilitators in the pathways to timely cancer diagnosis, and (2) perceptions of the barriers and facilitators of eHealth to support early cancer diagnosis (Multimedia Appendix 1). This study focused on the analysis of part (2) of the interview, aiming to identify infrastructural challenges, usability, and HCWs’ attitudes toward the use of eHealth to support symptomatic cancer diagnosis. The interview guide adapted concepts from The Technology Acceptability and Usability Survey [19] and the sociotechnical theory (STT) [20]. Technology Acceptability and Usability Survey is a survey developed to explore HCWs’ perspectives of using an mHealth tool in Nigeria; authors synthesized literature on mHealth usability and acceptability worldwide to guide the development of a tool intended to be more suitable for low-resource settings and tested its validity through consultation with a panel of clinicians and researchers. The STT is “an 8-dimensional model specially designed to address the socio-technical challenges involved in design, development, implementation, use, and evaluation of HIT [health information technology]” [20]; it describes how the interrelated and nonhierarchical dimensions compose a complex system that determines how HIT is used. It was intended to guide optimization of the social and technical factors in developing organizational systems by mapping the complex relationships between people, processes, and technological systems [20].

Following interim analyses, 4 clinical advisory group workshops were conducted (2 in each country) between March 18 and May 23, 2024. The participants were doctors and nurses working across all levels of care in South Africa and Zimbabwe. Preliminary findings were presented from the IDIs, as well as from a health facility audit and cross-sectional survey assessing the availability of services and infrastructure at the study sites. After presenting the findings, a facilitator-led discussion using a question guide was conducted, focusing on (1) key challenges in the pathway to diagnosis for breast, cervical, and colorectal cancer at each health care level, (2) best practices for managing each cancer across health care levels, and (3) requirements for eHealth adoption to support symptomatic cancer diagnosis. Workshops were audio-recorded and transcribed, and transcription notes were recorded during the workshop.

### Data Analysis

We used the Framework Method to analyze the interview data, a codebook method broadly part of thematic analysis [21,22]. The Framework Method uses an analytic framework to categorize and organize data using codes and includes five stages after collection and transcription: (1) familiarization, (2) coding, (3) developing an analytical framework, (4) applying the framework, (5) charting data into the framework, and (6) interpreting the data [21]. After familiarization, a codebook was developed collaboratively by 3 coders (KDA, NT, and Sunga Mzeche), guided by the 8 dimensions of the STT, which we used as our guiding framework. The coding framework was adapted at different stages of analysis as we became more engaged with the data, a flexible process commonly used in reflexive thematic analysis [23]. Each interview transcript was indexed by 2 of the 3 coders using NVivo (version 14; Lumivero). We compared codes, highlighted disagreements, and refined the codebook through discussion, with support from SES and FMW. Final themes were developed by KDA to derive meaning from the data and answer the research question. The transcripts from the workshops were analyzed thematically by VAS to categorize the content of the discussions and were then compared to the IDI findings.

### Ethical Considerations

All participants were required to provide written informed consent before interviews, in English or local languages based on their preference. Interviews took place during the participants’ working hours with permission from their employers, and none were compensated for participation. All transcripts were anonymized at the time of transcription, before being sent to the rest of the research team. Only the interviewers and transcriber were unblinded to the identities of the research participants, as this was unavoidable during data collection and transcription. Details, such as place of work, departments, and names of colleagues mentioned in the interviews, were anonymized in interview quotes in this paper to avoid possible identification of participants or departments by readers. Audio recordings and transcriptions were stored in a secure drive on the laptop of SD. SD shared anonymized transcriptions with KDA and NT through Microsoft SharePoint (Microsoft Corp), which required a 1-time PIN sent via their work emails each time a file was opened. This study was part of a broader study protocol funded by the National Institute for Health and Care Research (NIHR133231) and was approved by (1) the University of Cape Town, Faculty of Health Sciences Human Research Ethics Committee (HREC; reference: 664/2021 and HREC 892/2023), (2) the Joint Research Ethics Committee at the University of Zimbabwe (Joint Research Ethics Committee reference: 363/2021), and (3) the Medical Research Council, Zimbabwe (HREC reference: MRCZ/A/2831). All ethics committees approved this study involving human participants.

## Results

### Participant Characteristics

Fifty-six HCWs participated in the IDIs, including 26 from South Africa and 30 from Zimbabwe, of which 24 (43%) worked in primary care and 32 (57%) worked in secondary or tertiary care (Multimedia Appendix 2). The median age was 44 (IQR 34‐53) years, the median time in their role was 6 (IQR 2‐15) years, and most were female (n=38, 68%). Most participants (n=52, 93%) had a higher education degree (postgraduate degree, undergraduate degree, or diploma), 3 (5%) had Matric or O-levels, and 1 had a certificate. The participants included 34 (61%) nurses, 15 (27%) doctors, 3 (5%) medical managers, 1 health information officer, 1 clinical assistant, 1 data clerk, and 1 receptionist.

### Definitions and Current Access to Digital Tools

We defined terms for the concepts commonly identified in the data. “Digital tools” are mobile, computer, or web-based applications [24] that can be used on electronic devices such as mobile phones, tablets, or computers. Digital tools that have been designed specifically for health care purposes or adapted to be used for health care purposes are referred to as eHealth. eHealth and electronic devices were widely used in cancer health care services across the interviews and were more evident and advanced in South Africa than in Zimbabwe, and at secondary or tertiary levels of care compared to primary care. We defined “personal devices” as electronic devices owned by the individual and “provided devices” as electronic devices purchased and owned by employing institutions. The participants used a range of digital tools on either personal or provided devices, which were categorized into 3 groups (Table 1) based on their descriptions and published resources regarding specific tools. We refer to (1) “public-access tools” as digital tools available to the public on any device (personal or provided) connected to the internet (via Wi-Fi or cellular data) of which many were not designed for eHealth but can be adapted for eHealth purposes, (2) “partially restricted tools” were eHealth tools available on any device (personal or provided) connected to the internet but logins were restricted to subgroups of HCWs such as doctors or administrative staff, and (3) “restricted tools” were eHealth tools only accessible on hospital-based computers using a restricted login. Some HCWs were given “provided devices” with or without internet (Wi-Fi or cellular data) for use in work-related purposes. The provided devices were used to access “restricted tools” and “partially restricted tools”; however, access to “public-access tools” on the provided devices varied across cases. HCWs also used their personal mobile phones to access “public-access tools” or “partially restricted” tools.

**Table 1.** Types of eHealth used in health care are described in in-depth interviews.

Tool type	Description	Examples
Public-access tools	Anyone can download applications, create accounts, and use the tools from anywhere and on any personal (or some provided) device connected to the internet (ie, using Wi-Fi or cellular data) via mobile or web-based applications. Tools may or may not require payment for use. Tools were usually designed for non–health-related purposes but have been adapted for use in the health care setting.	Jotform (Jotform Inc) Google Calendar (Google LLC) Email WhatsApp (Meta)
Partially restricted tools	Tools that are available only to a specific subgroup of people or health care workers (eg, administration staff, doctors, or nurses) but can be accessed through any personal or provided device connected to the internet via mobile or web-based apps with a user login.	National Health Laboratory Service Vula Mobile (Mafami Pty Ltd)
Restricted tools	Tools that are only available on devices provided by the local health services (eg, facility-based computers) and have restricted access via a login and password. Internet access may or may not be required.	Clinicom [25] Impilo (Impilo, Inc) [26] TriMed (TriMed Inc) ePOC [27]

### Themes

#### Overview

Four core themes were developed to demonstrate the barriers and facilitators of digital tool use among HCWs to support symptomatic cancer diagnosis in South Africa and Zimbabwe. All coders suggested quotes for inclusion, and by consensus with the wider team, quotes that best encompassed the meaning of the themes were selected (Tables2^5^). Where possible, quotes were included from a spectrum of participants by region, health care level, and job role to demonstrate similarities or contradictions across participants. At the end of each theme, we describe how they relate to different aspects of the STT.

**Table 2.** Theme 1—the lack of reliable infrastructure hindered digital and eHealth tool use.

Subtheme	Quote
Lack of electricity and network or internet connectivity	”Because the back-up system is not reliable. Firstly, when we lose electricity in this town, we lose network coverage. And when we lose electricity, sometimes the computers don’t go on. Especially if the back-up doesn’t kick in. So that impacts our work in a negative way.” [Doctor, Eastern Cape, South Africa]. “Yeah, it’s okay but we need some power backup because at times there is no electricity. We can spend some 2‐3 days without electricity so this will disrupt the workflow especially when you are using EHR [Electronic Health Records].” [Nurse in charge, Harare, Zimbabwe].
Duplication of work	”Well, you see because I have a backup book, it doesn’t affect my work so much. You’re stuffed if you’re just on the computer. If you just electronically, it’s going have quite a bit of an impact on you. But if you have some old school backup system, then you just revert back to that system.” [Nurse, Western Cape, South Africa]. ”So, the problem is with loadshedding and often it’s offline or there’s something wrong and it needs an update or an upgrade. It’s frustrating because you then have to go back to a manual system.” [Medical manager, Western Cape, South Africa]. “When internet is down now you go to manual and it takes time. It increases the patient’s waiting time. For electricity at least we have the backup, but the backup is covering essential departments like suppose electricity goes out when the patient is on the table in theatre, or the patient is in ICU on a ventilator, so the generator should take over. …I think paper has served us well and even if we go technology, we are still supposed to promote this to fall back on.” [Nurse, Bulawayo, Zimbabwe]. ”…the electrical infrastructure in the country is very poor. …when the system is down, I can’t see anything and so that is a big draw back. If the whole system crushed, we would have a very big issue. …to be honest, we probably do not have adequate back up and so back up system redundancies, power back up, information back up. We do not have a cloud server. We do not have a hard drive.” [Clinical assistant, Harare, Zimbabwe].

**Table 3.** Theme 2—use of personal mobile devices and public-access tools allowed for digital and eHealth tool use at the expense of patient privacy and personal cost.

Subtheme	Quote
Use of personal devices or data	“But hence, I’m saying the network issues… You have to use your own network at some point to get your patient’s results… if you need to call for anything you need to use your phone and your airtime.” [Nurse, Eastern Cape, South Africa]. “I use my personal device all the time. This [my mobile phone] is my office. …Often the computers are down, so we need to check blood results and stuff. We often use our [personal] phones. Often it’s a bit faster because the internet and stuff is slow in the hospital.” [Doctor, Western Cape, South Africa]. “Sometimes when I want to call maybe another department, when the phone is down, I can use my phone. My personal phone.” [Nurse, Bulawayo, Zimbabwe]. Interviewer: “do you use any personally owned technological devices to support your clinical practice?” Interviewee: “Yes, if you need to research on anything you use your phone because no one is giving you data, no one is giving you…” [Nurse, Bulawayo, Zimbabwe]. “Most people here if they are told to go and have a scan or x-ray done, the results will come on a disk so we will need a laptop to be able to access the content of that disk. We use our phones to take pictures… for example maybe we would have forgotten our laptops, we go to our fellow colleagues with our phones and open on their laptops then use our phones then return with pictures and attend to the patients or having patients’ results being sent to our phones” [Nurse, Harare, Zimbabwe].
Affordability	“It’s [mobile data] not really affordable as such, but it has become a need, a requirement- daily requirement. It’s something that we have to accept that we need it.” [Medical superintendent, Bulawayo, Zimbabwe]. Interviewer: “if you’re using your personal, can you tell me more about how you go about using the internet and whether internet access is affordable for you.” Interviewee: “No, it’s not affordable and some of the searches they are limited because I will be saving my data and also for something else. I can’t just use it for clinical purposes, yet I have something to do at home. So, I will limit.” [Nurse, Harare, Zimbabwe].
Patient privacy	“Now the challenge there is that we have not instituted the whole privacy hyper. I do ask my patients beforehand, “Can I take a picture? Can I send it by WhatsApp?”, but that’s a very- has lots of holes. …I think there probably is going to be some regulation of that because abuse of that will come eventually. Most likely all patients will feel like they are not having confidentiality at some point.” [Clinical assistant, Harare, Zimbabwe]. “That’s what a lot of the doctors will do [use their personal devices], just out of sheer frustration. There are obviously issues of the POPI [Protection of Personal Information] Act and all of those things that become a problem, but for the patient’s sake that’s what they do. You’ll compete with others [to use a computer] or go to another ward. You probably end up using your own device.” [Medical manager, Western Cape, South Africa].
eHealth champions	“I once introduced a booking system where we use a calendar for booking the patients but it didn’t really materialise, it didn’t really work out. I had to be there for it to work out but it ended up, because I left that unit, it just, they stopped using it after I left. So, I think attitude towards technology is also important. So, for it to work.” [Doctor, Eastern Cape, South Africa].

**Table 4.** Theme 3—information, workflow integration, and access.

Subtheme	Quote
Theme 3.1—accessibility and workflow integration	
Restricted access (logins and insufficient devices)	“So, the problem with some of these databases and systems is that not everyone is allowed access.” [Doctor, Western Cape, South Africa]. “The availability of e-viewing devices in our institution is also limited…We have been provided with a computer. A single computer, which luckily sits in my office, in that whole gynae OPD [out-patient department]. But it’s just a computer. There’s no internet.” [Doctor, Eastern Cape, South Africa]. “If you don’t have enough computers, so sometimes you’ve got to compete and wait, the other guy has got to finish before you can start punching in your patient’s stuff. …Often there’s three or four EC [emergency center] doctors working that’s probably competing for one or two computers. That’s definitely an obstacle.” [Medical manager, Western Cape, South Africa].
Multiple tools with poor integration	”If there was one functional system that covered everything, a clinical working system. I don’t actually care that I access it from my own phone, but one clinical system where I could access notes from [hospital name], and they can access patient notes from [another hospital name], that would be a huge step forward.” [Doctor, Western Cape, South Africa]. “That’s probably one of the big problems is there’s no uniform simple central one system that we can all use and it makes it easier of the patient.” [Medical manager, Western Cape, South Africa]. “But ideally if the systems were linked countrywide, it would have been easier, but I don’t think they have that except this and even the ePOC- [electronic point of care] the HIV treatment. That one also has an electronic system where if you have got your medication here, it’s nationally linked.” [Doctor, Bulawayo, Zimbabwe]. “The only challenge is that it only shows here at [clinic name]. So, we wanted it to link with other clinics so that let’s say we have referred someone to [clinic name], those at [clinic name] will immediately see that we have sent someone.” [Nurse in charge, Harare, Zimbabwe].
Easy access to information and improved efficiency with digital tool use	”Oh, it’s quick. It makes things easier because you can see how well the patient has been taking treatment, and all that. You can see the viral loads. You can see everything about that patient. You can get all the information about where does she or he stays.” [Data clerk, Eastern Cape, South Africa]. “Oh, absolutely, definitely the benefits are that I can see the X-ray in any department that I am in.” [Clinical assistant, Harare, Zimbabwe]. “The benefits of using EHR [electronic health records] is at least the information is always there and the information is readily available, and it is also transmitted when it is supposed to be transmitted early.” [Nurse, Harare, Zimbabwe]. “For now, no, I quite enjoy it because you can just click, click, click, and then take… Because previously we had to take all the stickers and all the clients that we saw for the day just in case we have to go back for anything. So, it’s one massive book missing. So, everything is just computerised and it’s much better. I quite enjoy it because it’s just much easier.” [Nurse, Western Cape, South Africa]. “I think with the help of getting information. At the hospital we are not using but here we are using a system like Odoo [OnDemand Offer from OpenERP] system to capture clients’ records. So, I think it lessens the work. Where you are writing four pages, you can just use it on a one-page system. So, it saves time. Then it’s also efficient.” [Nurse in charge, Harare, Zimbabwe]. “The benefits. I think time. It doesn’t take much of your time plus storing data. Unlike paperwork, you may want it, but data will always be there. It’s a very fast and convenient system.” [Nurse in charge, Harare, Zimbabwe].
Theme 3.2—communication and uniformity	
Immediate bookings	“I’ve taken it over because they had a book with stickers in. …It’s all on a Google Calendar now. So, I can book a patient for a mammogram now. If you need a mammogram, I can book you one. … I can just go onto any computer, I can access my calendars. … So, it’s a little bit better.” [Doctor, Western Cape, South Africa].
Delayed bookings or responses	“When you refer someone, there’s always a bit of a delay also on the day, because the person on the other side might be busy with other things. So, when he responds to accepting the patient and giving a fair date, it might be two or three hours down the line. That also causes a bit of waiting for the patients on our side. Often, we have to let the patient go home and say, look, give me your phone number, we’ll call you back tomorrow, and that’s another additional step in the process.” [Medical manager, Western Cape, South Africa]. “It is not helpful to get back to someone [after a referral] the next day with a date or whatever. It needs to be an immediate response. …You’re going to suck another half an hour of my life trying to find the patient again, get them to answer their phone and tell them what date they have to be at Urology. I don’t have time for that. Or, maybe, they phone the patient, I don’t know, and that doesn’t sit well with me. So, the patient leaves me, and I don’t know if it’s sorted.” [Doctor, Western Cape, South Africa].
Lack of uniformity	”Not everybody uses the same system, so you often have to phone the switchboard or somebody else to get the right place to go to, especially if it’s not something that you deal with every day.” [Medical manager, Western Cape, South Africa].
Theme 3.3—eHealth value in practice	
Lack of patient benefit	”I’m very sceptical of technology because it’s just used to collect data with no benefit to the patient and that’s not helpful.” [Doctor, Western Cape, South Africa].
Time-consuming and requires effort to complete	”…there’s quite a lot of information that you require from people [on Vula]. Sometimes you just feel, this is not necessary. But you can understand, on the other hand, that some people just refer anything. If people were allowed… So, there’s got to be a way of asserting that this referral is the correct one. So, the more information they put in. So, it may be frustrating for them but this is the information you get.” [Doctor, Western Cape, South Africa]. “Your data capturing needs to be part of your daily routine otherwise people will not capture data. Forget about it… And that’s why I stopped using REDCap…REDCap is a research tool. It’s not an electronic patient record. It wasn’t designed for that, so you can’t do that with REDCap.” [Doctor, Western Cape, South Africa].

**Table 5.** Theme 4—digital health is expanding whether we like it or not.

Subtheme	Quote
Resistance to change and digital tool use	“There’s quite a bit of resistance with electronic apps. Not just with me. I mean, I am not comfortable with it, but I wouldn’t be resistant to it.” [Nurse, Eastern Cape, South Africa]. “I think, it’s fear. Fear of the unknown. But, I think, once the younger people get the hang of it. And change, people don’t like change.” [Nurse, Western Cape, South Africa].
Technology use among the younger generation	“So, when people are forced to, it is frustrating, and some people still don’t get it but I think it’s possible, especially if we get more and more of the younger generation.” [Doctor, Western Cape, South Africa].
Skepticism toward digital tool use in health care (reliability and feasibility)	“How comfortable, meaning, do I have the skill? Yes, I have the skill. But how comfortable am I in terms of, do I believe that these online mobile will reduce cervical cancer? I’m not convinced. Single-handedly, I’m not convinced.” [Doctor, Eastern Cape, South Africa]. “Everyone, when they said [Hospital name] now will pioneer with maternity they said let’s go paperless tomorrow and we just warned, it’s not as easy as that because we need to see how functioning it is and in parallel with the paper. We can’t just go paperless.” [Nurse, Bulawayo, Zimbabwe].

#### Theme 1: The Lack of Reliable Infrastructure Hindered Digital and eHealth Tool Use

Most participants reported that the lack of reliable infrastructure hindered the use and expansion of digital tools, both through HCWs’ ability to use them (theme 1) and the impact it had on their attitudes towards them (theme 4), and was worse in rural areas than in urban areas. This was particularly relevant to “restricted tools” on provided computers because many reported they were vulnerable to frequent power cuts, poor internet or network connectivity, and insufficient devices for the number of HCWs who needed them. Some HCWs from South Africa noted that challenges due to electricity cuts (referred to as “load-shedding”) were partially solved by using generators; however, 2 participants noted that there were still brief interruptions in power supply during the changeover from electrical to generator energy that resulted in computers resetting and data loss. Whereas in Zimbabwe, generators were either unavailable or had limited power that was prioritized for essential services, such as for the use of ventilators and to operate theaters. Owing to the unstable power supply and HCWs’ fear of losing data, many continued to use backup, paper-based systems alongside digital in both countries, thus duplicating their work. With regard to STT, theme 1 (Table 2) draws upon dimension 1: hardware and software computing infrastructure, which “focuses on the hardware and software required to run applications” [20] as well as challenges within dimension 2: the human-computer interface*,* which acknowledges how humans engage with eHealth within their clinical context and workflow, and if or how they can engage while providing patients with the care they need when they need it, thus incorporating elements of dimension 5: workflow and communication.

#### Theme 2: Use of Personal Mobile Devices and Public-Access Tools Allowed for Digital and eHealth Tool Use at the Expense of Patient Privacy and Personal Cost

In South Africa, most participants reported using provided devices to access digital tools, compared to few in Zimbabwe. Significant barriers to using provided devices included restricted access to the internet, being unable to use “public-access tools” such as Google Calendar, and having insufficient electronic devices. In contrast, using personal devices to access digital tools posed fewer restrictions, and most HCWs in both countries had access to personal mobile devices and used them for work. However, many noted that internet access was not provided, and cellular data was unaffordable.

Many participants from South Africa and 1 in Zimbabwe highlighted that “public-access tools” gave them the autonomy to choose how to adapt tools to the specific needs of their departments (such as Jotform [Jotform Inc] and Google Calendar [Google LLC] to book appointments). Being publicly available, these tools also facilitated cross-departmental and interfacility collaboration and communication without the need for approval from hospital management. While the accessibility of “public-access tools” was a major advantage, using these tools (particularly on personal devices) raised concerns regarding the protection of patient data. HCWs frequently used WhatsApp (Meta) to communicate, and in some instances, photos and patients’ personal details were shared between HCWs despite a limited understanding of how the data were stored or protected. “Partially restricted tools” brought about fewer data security flags as logins were still required and data were not stored on devices. HCWs also appreciated that they could use “partially restricted tools” on personal devices, particularly as hospital-based computers were unreliable or unavailable. Some HCWs drew strict boundaries on using their personal devices for work, whereas others highlighted it as the only access they had to some digital tools and accepted the personal financial cost.

In some areas, departmental leads took the initiative to use “public-access tools” in their daily practice to suit the needs of their departments. While these tools were acknowledged as playing an important role in improving clinic efficiency or daily practice, having a single “champion” who oversaw the use of the tool meant its long-term sustainability was subject to their presence, even in the short term, such as when taking sick leave. Despite these challenges, the accessibility of “partially restricted” and “public-access” tools was a great benefit, compared to “restricted tools” which were governed by hospitals or regional health departments and vulnerable to infrastructural barriers.

As the use of personal devices and data resulted from the lack of reliable infrastructure, this theme relates to dimension 1 of STT: hardware and software computing infrastructure as well as dimension 2: clinical content, which “includes everything on the data-information-knowledge continuum that is stored in the system” as it pertains to the sharing and storage of health data; dimension 6: internal organizational policies, procedures, and culture, which incorporates the control of budgets and procedures (or the lack thereof) to use eHealth, and thus the lack of budgets and financial constraints that led to the use of personal devices; and dimension 7: external rules, regulations, and pressures, which refers to external forces that “facilitate or place constraints on the design, development, implementation, use and evaluation of health information technology,” as personal device use and storage or sharing of data on personal devices hinders access to and evaluation of eHealth. The subtheme “eHealth champions” also relates to dimension 4: people, as this represents the “humans involved in the design, development, implementation, and use” of eHealth, including how systems make users think and feel.

#### Theme 3: Information, Workflow Integration, and Access

In both countries, many HCWs noted multiple digital and eHealth tools in use that all served different purposes, each with their own login details, and some restricted to certain cadres of staff. This lack of tool integration hindered workflow, and HCWs demonstrated frustration with the need to have multiple passwords. HCWs advocated for the integration of different systems with access using a single login on one app to provide all the information they needed about their patients. Many tools were designed to improve the efficiency of work, and participants appreciated these functionalities, such as the use of an app to view x-rays which had “restricted access” instead of fetching physical copies from the radiology department, as well as the ease of access to patient information that was stored digitally compared to paper records, which could frequently get lost. However, insufficient devices or computers located in areas far away from consulting rooms disrupted workflow while they went to find available computers, diminishing the value of the tools.

Specific to South Africa, some HCWs noted that tools used for referrals or appointments did not allow for instant engagement or immediate responses. Vula (Mafami Pty Ltd) is used in South Africa for communication between clinicians to discuss, refer patients, or book appointments. Some participants noted that responses were often received hours or days later, interrupting consultations; patients had to be sent home while awaiting feedback and were unable to get hold of staff via telephone. Other tools allowed for instant bookings (eg, Jotform, Google Calendar, and Clinicom), and participants noted this as a significant advantage over other systems. Some tools were used consistently across regions, whereas others were specific to single departments or hospitals. The lack of uniformity created complexity as this required knowledge of the correct system for referral or appointments for every department, although 1 clinician took the time to provide feedback to referrers when they attempted to use the “wrong” route for referral.

Some participants in South Africa felt the tools were not designed to benefit patients, and they were being “used to collect data” (Doctor, Western Cape, South Africa) because paper-based systems continued to be the primary method for documenting patient care. If tools had limited patient benefits and took additional time from consultations to complete, HCWs were reluctant to use them. In comparison, tools that replaced paper systems endured infrastructural challenges, and they frequently “switched back to manual” when electronic systems were down (theme 1).

Theme 3 incorporates cross-cutting dimensions of the STT, including dimension 2 (described above) and dimension 5: workflow and communication, which “accounts for the steps needed to ensure each patient receives the care they need, and must be modified to adapt HIT or the HIT system must change to match the various workflows identified” [20].

#### Theme 4: Digital Health Is Expanding Whether We Like It or Not

Many HCWs were familiar with the use of digital tools in their day-to-day practice, and for some, their past negative experiences led to skepticism toward their use, particularly regarding their reliability and feasibility in practice. The distrust in the reliability of digital tools stemmed from the unsuitable infrastructure for their use (theme 1), lack of training among staff, and the wealth of other unresolved systemic issues that some were convinced would not improve with the use of digital tools or eHealth. One participant in South Africa highlighted their skepticism in eHealth as the answer to “reducing cervical cancer” because they believed basic services and structural challenges underlying the poor cervical cancer outcomes needed to be resolved first, such as training and competence among HCWs.

Some HCWs acknowledged their own discomfort in using digital tools or eHealth but recognized the inevitable trajectory of shifting away from paper-based systems and accepted that they would need to adapt. Particularly evident among the older generation, some HCWs demonstrated a strong resistance to change, highlighting that they were too old to learn to use digital tools or eHealth and felt it was the responsibility of the younger staff to learn, teach others, and facilitate wider adoption within the health system. Others also highlighted the need to include training to use digital and eHealth tools and better support for troubleshooting as part of further integration and adoption. Training is discussed in more detail in a parallel paper [28].

This theme is predominantly related to dimension 4 of the STT: “people,” representing all the people involved (developers, users, trainers, clinicians, and patients) in all aspects of design, and includes the way systems help users to think and how it makes them feel [20]. The theme also draws on aspects of dimension 3: “workflow and communication,” as the availability, functionality, and efficiency of these systems influence how they engage with and feel about eHealth.

### Credibility Checking

A total of 26 doctors and nurses (12 in South Africa and 14 in Zimbabwe) from all levels of care participated in the clinical advisory group workshops across both countries. Themes and recommendations derived from the workshops (Multimedia Appendix 2) were concordant with the IDI findings, and no additional themes were generated. As reflected in the main themes from the IDIs, the workshop participants highlighted the need to design tools that are suitable for contexts with limited electricity and network connections. Similarly, they also highlighted the need to build on existing systems as there are currently too many tools with poor integration, resulting in the repetition of work, thus devaluing their function.

## Discussion

### Principal Findings

This study explored HCWs’ experiences of using eHealth to support symptomatic cancer diagnosis and the requirements for future adoption and expansion. While many previous studies explored HCWs’ experiences of eHealth generally [29] and for cervical cancer screening in sub-Saharan Africa [30], few quality studies have explored eHealth use to support symptomatic cancer diagnosis. This study adds important insights to the limited body of literature and offers recommendations for the design or adaptation of eHealth to support symptomatic cancer diagnosis in Southern Africa (Textbox 1). These are broad, and prioritization will depend on the contextual needs and the specific tool being designed. The implementation of these recommendations requires a coordinated approach between tool developers, users, and the wider health care infrastructure. This could be facilitated by developing a roadmap to improve the use of eHealth in Southern Africa.

Textbox 1. **Recommendations for future digital health tool development and implementation in Southern Africa.**

**Digital tool and implementation-specific recommendations**

- Flexible accessibility—for example, “partially restricted” tools.
- Interoperability—access to information by different actors (health care workers, providers, or auditors).
- Single tool to access all patient information or tool integration across multiple tools.
- Single login for all tools.
- Usable with limited or no internet connection.
- Adheres to local or national health data security recommendations.
- Training to use digital tools and support for troubleshooting.
- Desirable tool functions—immediate bookings, user autonomy, easy access to information, or communication across departments.
- The tool replaces the paper system and has an adequate back-up system (ie, no duplication).
- The tool is used for patient care as the primary aim and data capture as the secondary aim.
- The tool should improve the efficiency of work and minimize additional time from consultations.
- Easy to use—accessible with limited digital proficiency.

**Infrastructural recommendations**

- Uniformity in systems used across health care facilities and levels within regions, supported using guidelines to identify clear pathways for referral or digital tool use.
- Involvement of higher managerial levels to oversee use and sustainability.
- Use of “tool champions” with contingency plans for staff changes.
- Information technology support for ongoing maintenance and sustainability.

### Comparison With Existing Literature

While we purposively sampled individuals who were exposed to people with symptomatic cancer, cancer is a rare disease with vague and common symptoms; thus, participants spoke more broadly of their experiences using eHealth rather than specifically relating to symptomatic cancer. In our parallel paper, we noted that most participants came across people with possible symptoms of cancer on a regular basis, while rarely seeing someone with cancer, and never finding out if they had cancer after they were referred to secondary care [28]. Thus, we concluded that the experiences of the participants were in managing possible symptoms of cancer, rather than in managing individuals with a known or likely cancer diagnosis.

Our findings demonstrate that eHealth is used in a variety of clinical settings. However, there are widespread reports of infrastructural challenges hindering eHealth use, which may have fueled the observed skepticism about its potential to improve cancer outcomes. In a systematic review of barriers and facilitators to eHealth use among HCWs worldwide [29], infrastructural challenges were similarly highlighted as a key barrier. With South Africa and Zimbabwe having fewer resources than the countries in the review, the investment required to prepare Southern Africa for eHealth expansion will be greater than that in other regions worldwide. The poor infrastructure and distrust in the reliability of eHealth led to parallel use of paper-based systems; thus, the additional effort required to use eHealth may have outweighed the potential efficiency value. Similarly, HCWs’ skepticism and the perception of usefulness were noted as barriers in other studies [29], although duplicated paper-based systems were not mentioned. Nevertheless, several participants recognized the adoption of eHealth as inevitable and were open to accepting the changes needed, and many expected the younger and more tech-savvy staff to facilitate the transition into the digital era.

Many HCWs adopted “public-access” tools such as WhatsApp to develop informal solutions to the structural challenges they experienced, which required the use of personal devices and data, at a personal financial cost and to patient privacy. This raises ethical concerns, and policy makers should consider adapting local guidelines to reflect recommendations from the World Health Organization’s policy on Personal Data Protection, as well as ensuring adequate implementation in practice [31]. Personal device use was commonly observed in other studies among HCWs attempting to tackle systemic gaps in health care services [32,33]. While personal devices benefit from being more accessible, advocating for their use risks shifting the cost burden from health systems to HCWs [34]. Personal device use also makes standardization of practice challenging, and the informal pathways may disrupt or replace formal systems [32]. Interoperability, the access to and sharing of health information between clinicians, health care organizations, and providers, is a key principle of digital health [35]. With HCWs using “public-access” tools, not governed by formal systems, information may only be accessible to individuals’ personal devices, thereby disabling continuity of care and the ability to use the information for auditing and quality improvement. The use of “restricted tools” also comes with challenges. Relying on low-resourced facilities to provide electronic devices could mean that there is never enough momentum to roll out a new digital intervention or evaluate its impact. Future eHealth designs should aim to ease the flexibility of access, such as the devices they can be used on, while prioritizing data security and ensuring interoperability. This may mean adopting some aspects of “partially restricted tools” such as accessibility on personal devices, better data security through login access limitations, and the ability to share information between different health actors or systems. The ethical and legal challenges of data security when HCWs use personal devices for work have been highlighted in previous studies [29,32], although solutions were not discussed and require further exploration, and stronger local and international guidance and policy. In our study, HCWs noted that eHealth disrupted workflow and consultations. Other studies similarly showed that HCWs receiving phone calls during consultations were disruptive and were seen as unprofessional by patients [36]; however, little is known about how this may play out when HCWs use mobile phone apps, and this warrants further exploration.

### Implications for Public Health and Future Directions

There is growing debate about the impact of eHealth use on climate change. The value it has in tackling climate change lies in the reduced use of transport from virtual services, such as telemedicine replacing face-to-face consultations, and electronic record keeping and communication, such as prescriptions and referrals, that reduce the need for paper-based systems [37]. However, eHealth can also contribute negatively through the production of electronic waste and increased consumption of electricity [38], which in many low-resource settings is reliant on burning fossil fuels, potentially mitigating the value of eHealth in reducing emissions [37]. Furthermore, in South Africa and Zimbabwe, electricity is limited, and its use is prioritized for essential systems such as operating theaters and ventilators, among many others. While a switch to renewable energy production could create an adequate and sustainable electricity supply, it requires major investment and development extending beyond the health sector [39].

In interviews, HCWs noted that internet access was limited. Future eHealth interventions should explore the possibility of no- or low-data usage apps or those that can be used offline and uploaded retrospectively. Mobile data is exceptionally expensive in sub-Saharan Africa, with Zimbabwe having the most expensive data worldwide (43.75 US $/GB) [40], while other countries in the region have strikingly lower data costs despite similar HDIs, such as Malawi (0.39 US $/GB) and Mozambique (0.78 US $/GB) [40]. Governments in South Africa and Zimbabwe should consider strategies to reduce the excessive cost, provide free or subsidized internet, and invest in infrastructure to improve access. eHealth has the potential to improve health care access to remote communities; however, those in more resource-limited and rural areas often lack internet and electricity [34]. Unless these issues are addressed, eHealth could inadvertently worsen health inequities. The United Nations Development Programme recommends guidance on the use of eHealth and human rights, highlighting strategies to promote inclusive digital transformation and minimize the risk of exacerbating health inequalities [39], such as promoting open-access eHealth and facilitating infrastructural development.

Before developing eHealth interventions for early cancer detection, the availability of resources for the next steps in the cancer pathway should be assessed. South Africa has mostly free or subsidized health care services in the public sector [41], but in Zimbabwe, a significant burden of costs falls on patients [42,43]. Without improving access to services in the next steps in the cancer pathway, such as for investigation, diagnosis, and treatment, eHealth interventions to improve early detection may be futile and ethically questionable. There is already an overwhelming number of eHealth interventions in use, and policy makers should consider better integration or adaptation of existing tools to streamline current practices before embarking on new developments.

### Strengths and Limitations

This study’s involvement of a diverse sample of participants from multiple levels of health care within rural and urban regions and the triangulation of IDI findings in workshops strengthens its credibility in reflecting the views of its participants.

This study had a few limitations. First, the interviews in Zimbabwe were mostly conducted in local languages, which risked a loss of depth or misinterpretation of meaning during translation to English. This could explain why we were able to highlight stronger patterns and draw more conclusions from the South African context than the Zimbabwean. We attempted to mitigate this risk by (1) using multilingual interviewers rather than an interpreter, (2) having the multilingual interviewers check the accuracy and completeness of the translation to English during transcriptions, and (3) adapting the interview guide after debriefing. Second, the use of only 2 countries in Southern Africa limits the generalizability of findings to the region more broadly. Due to budget constraints, we were unable to expand the research beyond these 2 countries. However, South Africa and Zimbabwe have varying HDIs, and the differences and similarities observed across them remain valuable. Third, despite purposively sampling for these characteristics, we were unable to draw comparisons across provider type and years of experience, as the differences or similarities were not strong enough. Lastly, participants spoke broadly about their experience of using eHealth, while our study aimed to focus on symptoms of possible cancer. We are limited in the ability to generalize findings to symptomatic cancer, which is rarely seen. Nevertheless, the lack of evidence for symptomatic cancer specifically highlights the need for further investigation.

### Conclusions

eHealth has the potential to improve access to care in Southern Africa, such as to support the management of people presenting to primary care with symptoms of possible cancer. However, to capitalize on its potential benefits, future interventions or adaptations to existing tools need to consider addressing infrastructural challenges, designing tools to suit the user’s needs, and being well integrated into the workflow. The need for essential resources to adopt eHealth, such as electricity, in low-resourced areas should be weighed against the need in competing priority areas, such as operating theaters, particularly as the long-term resource availability is threatened by global warming. As energy production in these regions is largely reliant on fossil fuels, eHealth risks impact the climate negatively. The findings of this study can be used to guide eHealth development or adaptation and the design of more contextually suitable implementation strategies.

## Supplementary material

10.2196/68412 Multimedia Appendix 1 Interview guide.

10.2196/68412 Multimedia Appendix 2 Findings from clinical advisory group workshops.

10.2196/68412 Checklist 1 SRQR checklist. SRQR: Standards for Reporting Qualitative Research**.**

## Abbreviations

AWACAN-ED: African Awareness of Cancer & Early Diagnosis
HCW: health care worker
HDI: Human Development Index
HIT: health information technology
HREC: Human Research Ethics Committee
IDI: in-depth interview
mHealth: mobile health
STT: sociotechnical theory
