# Supplementary material for: Health Care Workers’ Perspectives on the Barriers and Facilitators to Digital Health Technology Use to Support Symptomatic Cancer Diagnosis in Southern Africa: Qualitative Study
Source: J Med Internet Res. 2025 Jul 24;27:e68412. doi: 10.2196/68412 (PMC12289292; doi:10.2196/68412)
Supplement: Multimedia Appendix 2 [file jmir-v27-e68412-s002.docx]

**Clinician workshops**

**Table S1.** Overview of roles of clinicians participating in workshops.

| Country | South Africa | | Zimbabwe | |
| --- | --- | --- | --- | --- |
| Date | 18-Mar | 23-May | 18-Mar | 26-Mar |
| Location | Cape town | Cape Town | Harare | Bulawayo |
| Participant # | 6 | 6 | 7 | 7 |
| Job roles | PHC & Specialists | Specialists | 3 PHC, 4 Specialists | 3 PHC, 4 Specialists |
| Cancers | Breast & cervical | Colorectal | Breast, Cervical & Colorectal | Breast, Cervical & Colorectal |
| Clinician roles | - Family physicians - Endocrine & Breast Surgeon - Gynaecological Oncologist - Gynaecologist | - Colorectal surgeons - Registrars - Oncologist | - Gynaecological Oncologist - Surgical Gastro-oncologist - General Surgeon (Breast) - Medical Officer - Clinical Oncologist | - Gynaecologist - Surgeon, Breast Specialist - General Surgeon, Colorectal Specialist - General Practitioner - Medical Officer - Oncologist |
| *PHC = primary healthcare* | | | | |

**Table S2.** Overview of themes derived from the clinician workshops.

| Themes | Summary |
| --- | --- |
| HCW Training & education | - The nurses are first point of contact, seen as key & have a wide variety of training and experience. - Across cancers, training needed to recognise symptoms and know next steps for all HCWs. - Primary care HCWs report not being aware of referral pathways and general lack of knowledge of services available – need better information sharing. - Concerns about value of current clinical breast examinations and breast health policy - not properly implemented |
| Feedback to Primary Healthcare | - More feedback and communication back to primary is needed - Primary care keen to have feedback on whether referrals were appropriate - seems to happen in Gynae in SA now - Request for feedback and statistics to be incorporated into a future tool. |
| System/Approach changes to improve pathway | - Better use of existing resources and more resource for cancer is needed. System currently overburdened - Communication issues and lack of electronic data capture - An improved referral pathway through the system generally felt will reduce delays - Biopsies mentioned with differing views - SA view to centralise biopsies to speed up process, Zim view to decentralisation to reach more people & train/upskill to do biopsies - Difference reported between local & rural patients - seen as a failure of the Health System |
| Referral support, templates, checklists & guidelines | - View that a future tool should have referral processes built in & facilitate referral (example given of breast cancer system in SA that work well & has referral process built in) - Checklists & reminders of things to check/symptoms to be aware of should be included - an existing prompt list for TB in Zim mentioned - Pointers and information about what options are available and where to send the patient next to be included. - Algorithms built in to say what to do with different patients - New guidelines need to be context-specific – NICE & NCCN guidelines inappropriate for developing countries |
| Electronic / Paper / Offline | - *Info from Zimbabwe only in this theme* - View that the tool should be electronic, but must be available offline when don't have data or internet connection - Concern expressed that an electronic-only tool would exclude some people and view that both an electronic and paper version would be needed to cover |
| Tool features / acceptability | - View that it would be difficult to develop a tool for multiple cancers but that it would be useful - Any tool must make life easier for the HCWs to be accepted and must speed up the patient's pathway - Co-development recommended with piloting & input from language experts - view that it should target use by doctors and nurses - Desire to build on and improve on what is already out there - *Jotform* mentioned and discussed as to whether this could work in a paper-based system. The *Vula* app in SA was mentioned - the dominant view was that this was unpopular since it is seen by some as 'a tool to delay patients', however others had better experiences of it |
| Screening | - SA: screening is not working currently & focus should be on raising awareness - Zimbabwe: more screening is needed - there is not enough and what is there is inadequate |
| Patient Awareness & Education* | - General agreement that there is a lack of awareness of symptoms and risk factors which leads to late presentation. - Suggestions of awareness and prevention campaigns targeting schools, community centres, workplace, and health facilities - agreement with the - Community workshops. Also mention of village health workers working in the community. - Low health literacy seen as a barrier - people failing to understand the gravity of their situation. |
| Patient fear, stigma & beliefs* | - Patients believe cancer is a death sentence and fear of cancer is generally acknowledged. - Colorectal cancer patients additionally fear colonoscopy, surgical procedures and colostomy. - Symptoms 'not pleasant', deterring people from coming forward - Religious and cultural beliefs, use of alternative medicine, perceived treatment cost and lack of faith in the health system mentioned as barriers - Education, counselling and building trust between the community & HCWs seen as the way forward. |
| Costs & Transport* | - Costs and transport issues mentioned in all workshops as barriers Reports of funds running out and people not being able to pay for treatment after diagnosis. |
| **Caveat: these are HCW’s assumptions of patient barriers/facilitators to early diagnosis, rather than the perspectives of patients; HCW = healthcare worker; NICE = National Institute for Health and Care Excellence; SA = South Africa* | |

**Table S3.** Distribution of interview participants across the four 4 regions.

| Country and region | | Primary care | Secondary or tertiary care | Total by region |
| --- | --- | --- | --- | --- |
| South Africa | |  |  |  |
|  | Western Cape | 5 | 8 | 13 |
|  | Eastern Cape | 7 | 6 | 13 |
| Zimbabwe | |  |  |  |
|  | Harare | 9 | 9 | 18 |
|  | Bulawayo | 3 | 9 | 12 |
| Total | | 24 | 32 | 56 |
